# *Enterocytozoon bieneusi* in donkeys from Xinjiang, China: prevalence, molecular characterization and the assessment of zoonotic risk

**DOI:** 10.1186/s12917-020-02409-0

**Published:** 2020-06-15

**Authors:** Aiyun Zhao, Ying Zhang, Wen Wang, Bo Jing, Jinming Xing, Dayong Tao, Wei Zhao, Meng Qi

**Affiliations:** 1 College of Animal Science, Tarim University, Tarim Road 1487, Alar, 843300 Xinjiang China; 2 Department of Parasitology, Wenzhou Medical University, Wenzhou, 325035 Zhejiang Province China

**Keywords:** *Enterocytozoon bieneusi*, Zoonotic, ITS region, Genotype, Donkey

## Abstract

**Background:**

*Enterocytozoon bieneusi*, a zoonotic pathogen, has the potential to infect both immunocompromised and immunocompetent humans. It is found in large number of animals; however, not much is known regarding its prevalence in equine animals, particularly donkeys. This is the first molecular epidemiological evaluation of *E. bieneusi* in 178 free-ranging donkeys from five countrysides; and 502 farmed donkeys from 18 farms in 12 cities of Xinjiang, China by Nested PCR.

**Results:**

*E. bieneusi* was detected in 2.5% (17/680) donkeys, with 2.6% (13/502) in farmed and 2.2% (4/178) in free-ranging ones. Sequence analysis identified eight ITS genotypes, all belonging to zoonotic Groups 1 or 2, including six known genotypes: horse1 (*n* = 5), D (*n* = 3), NCD-2 (*n* = 3), BEB6 (*n* = 2), BEB4 (*n* = 1), and NIAI (*n* = 1); and two new genotypes: XJD1 (*n* = 1) and XJD2 (*n* = 1).

**Conclusions:**

This is the first report confirming the presence of *E. bieneusi* in donkeys in Xinjiang, China, and indicates the possibility of zoonotic transmission of this pathogenic parasite.

## Background

Microsporidia are obligate, spore-forming, intracellular pathogens, comprising more than 200 genera and 1500 species that are infectious to a numerous animals (both vertebrates and invertebrates) [1]. Of these, 17 species are known to be infectious to humans. Amongst these, *Enterocytozoon bieneusi* (*E. bieneusi*) is prevalent in humans and is known to cause life-threatening infections in people with a weak immune system [2]. Due to the global increase in the number of cases of *E. bieneusi* infection, it has been classified as an emerging infectious disease [3]. This parasite has also been identified in animals as well as water supplies, supporting the possibility of zoonotic infections with water contact acting as the transmission vehicle for the parasite [4–6].

Genotyping studies on the polymorphism of the internal transcribed spacer (ITS) region have revealed genetic mutations in *E. bieneusi* isolated from both domestic and wild animals, humans, and surface water [1, 2, 4, 7]. Currently, phylogenetic analysis has clustered the 470 known genotypes of *E. bieneusi* into 11 genetically-isolated groups [3]. Groups 1 and 2 include most humans and zoonotic genotypes, and thus, are important for public health, while Groups 3 to 11 constitute those genotypes that have adapted to specific hosts or wastewater, suggesting a reduced risk to public health [3]. Epidemiological studies focusing on genotyping the *E. bieneusi* extracts from those animals that have a higher probability of human contact would expand our current knowledge on the (a) prevalence of human microsporidiosis and (b) the zoonotic transmission of *E. bieneusi*.

There is a scarcity of data on the prevalence of *E. bieneusi* in equine animals, particularly donkeys [8–14]. Based on historical data, for the last 5000 years in China, donkeys, with a population exceeding 11 million, have been used for carrying heavy loads or for draught work in transportation/agriculture. They are raised for milk, meat, and Ejiao, a traditional Chinese medicine. Xinjiang Uygur Autonomous Region (hereafter referred to as Xinjiang) is the birthplace of China’s donkeys, and nearly a million donkeys living here. However, there is a scarcity of data on the prevalence of *E. bieneusi* in those animals from Xinjiang [13].. Herein, we evaluated the possibility of zoonotic transmission of *E. bieneusi* along with its prevalence in free-ranging and scale-farmed donkeys in the Xinjiang in China.

## Results

### Prevalence of *E. bieneusi* in donkeys

Six hundred and eighty fecal samples from donkeys were analyzed using nested-PCR to study the prevalence of *E. bieneusi*. We found *E. bieneusi* in 2.5% (17/680) of donkeys, with 2.6% (13/502) in farmed animals and 2.2% (4/178) in free-ranging animals. For the farmed donkeys, Yopurga (7/103; 6.8%) showed the highest infection rate of *E. bieneusi*, followed by Khorgas (1/20; 5.0%), Karakax (4/88; 4.5%), and Bohu (1/79; 1.3%). Other sampled farms showed no cases of *E. bieneusi* infection. For free-ranging donkeys, *E. bieneusi* was identified at two collection sites with infection rates of 5.9% (2/34) in Zepu and 3.1% (2/64) in Yecheng. No *E. bieneusi* infection was detected at the other three sites (Table 1). No statistically significant difference in the infection rates was observed between young and adult donkeys, which had a prevalence of *E. bieneusi* as 2.4 and 2.8% respectively, (*p* > 0.05). The study did not include young free-ranging donkeys (Table 2).

**Table 1:** Prevalence and genotype distribution of *Enterocytozoon bieneusi* in in donkeys in Xinjiang

Feeding pattern	Collection site	No. Positive/No. Samples (%)	*E. bieneusi* genotype (n)
Farmed	Alaer	0/54	–
	Barkol	0/11	–
	Bohu	1/79 (1.3)	BEB6 (1)
	Gongliu	0/21	–
	Huocheng	0/20	–
	Khorgas	1/20 (5.0)	BEB4 (1)
	Karakax	4/88 (4.5)	NCD-2 (3), XJD2 (1)
	Pishan	0/41	–
	Qitai	0/16	–
	Turpan	0/17	–
	Yuli	0/32	–
	Yopurga	7/103 (6.8)	horse1 (5), D (2)
Subtotal		13/502 (2.6)	horse1 (5, NCD-2 (3), D (2), XJD2 (1), BEB4 (1), BEB6 (1)
Domestics	Akqi	0/11	–
	Barkol	0/21	–
	Pishan	0/48	–
	Yecheng	2/64 (3.1)	BEB6 (1), NIA1 (1)
	Zepu	2/34 (5.9)	D (1), XJD1 (1)
Subtotal		4/178 (2.2)	D (1), XJD1 (1), BEB6 (1), NIA1 (1)
Total		17/680 (2.5)	horse1 (5), D (3), NCD-2 (3), BEB6 (2), BEB4 (1), XJD1 (1), XJD2 (1), NIA1 (1)

**Table 2:** Prevalence and distribution of *Enterocytozoon bieneusi* genotypes according to feeding pattern and age in donkeys in Xinjiang

Feeding pattern/ Age	No. Positive/No. Samples (%)	*E. bieneusi* genotype (n)
free-ranging	4/178 (2.2)	BEB6 (1), D (1), XJD1 (1), NIA1 (1)
Scale farming	13/502 (2.6)	BEB4 (1), BEB6 (1), D (2), horse1 (5), XJD2 (1), NCD-2 (3)
< 1 years	7/296 (2.4)	D (2), XJD2 (1), horse1 (1), NCD-2 (3)
> 1 years	9/323 (2.8)	BEB6 (2), D (1), XJD1 (1), horse1 (4), NIA1 (1)
Unclear	1/61 (1.6)	BEB4 (1)

### *E. bieneusi* genotypes in donkeys

We identified eight *E. bieneusi* genotypes in this study, which included six known genotypes: horse1 (*n* = 5), D (*n* = 3), NCD-2 (*n* = 3), BEB6 (*n* = 2), NIAI (*n* = 1), and BEB4 (*n* = 1), and two new genotypes: XJD1 (*n* = 1) and XJD2 (*n* = 1). Both new genotypes: XJD1 (MN174117) and XJD2 (MN174120) were found to be closely associated with genotypes BEB4 and O, with one and two nucleotide variations, respectively. Amongst the genotypes, horse1, BEB4, XJD2, and NCD-2 were found in farmed donkeys; XJD1 and NIA1 were found in free-ranging animals; genotypes BEB6 and D were identified in both feeding groups.

### Phylogenetic analysis

**Fig. 1:**
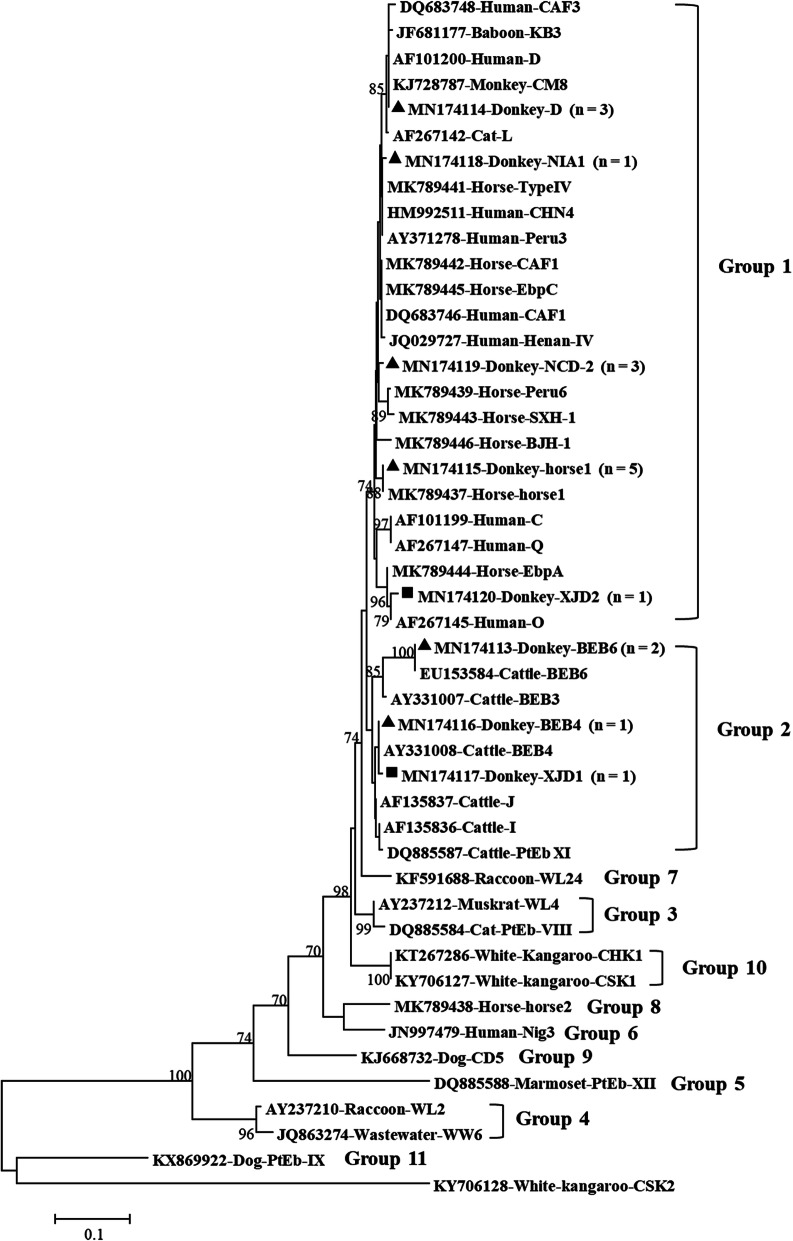
ITS sequence-based phylogenetic tree. Phylogenetic relationship between the known (GenBank) and identified (this study) *E. bieneusi* genotypes were identified through an NJ analysis based on the Kimura two-parameter model. Sequence detection was performed based on its host origin, accession number, and the designated genotype. The branches show the percent bootstrapping values from 1000 replicates. Outgroup classification comprised of the *E. bieneusi* genotype CSK2 (KY706128) from white kangaroo. The filled triangles and squares indicate known and novel genotypes, respectively

Phylogenetic analysis showed that group 1 genotypes were horse1, D, NIA1, NCD-2, and XJD2; and group 2 genotypes were BEB6, BEB4, and XJD1 (Fig. 1).

## Discussion

Previous studies have reported that in equine animals, the rates of infection of *E. bieneusi* fall in the range of 1.6 to 30.9%. However, there is a scarcity of data on the prevalence of *E. bieneusi* in donkeys (Table 3) [8–14]. Globally, only two studies, based in Algeria and China, have identified *E. bieneusi* in donkeys with infection rates of 1.6% (2/124) and 5.3% (16/301), respectively [8, 13]. Here, we reported the prevalence of *E. bieneusi* to be 2.5%, which was higher than 1.6%, which was reported in Algeria and lower than the 5.3% previously reported in China [8, 13]. These differences could be attributed to geographical factors, variations in feeding density, sample sizes, and differential management systems. In this study, the prevalence of *E. bieneusi* in farmed and free-ranging donkeys were 2.6% (13/502) and 2.2% (4/178), respectively and no statistical differences in infection rates were observed between them. However, in fact, the present study did not include any young free-ranging donkeys, only young farmed donkeys, which might have skewed the infection rate findings.

**Table 3:** Prevalence and genotype distribution of *Enterocytozoon bieneusi* in equines worldwide

Host	Country	Positive no. / total no. (%)	Genotype (no.)	Reference
Horse	Algeria	6.8 (15/219)	horse1 (6), CZ3 (2), D (1), horse2 (1), Unknown (5)	[8]
	Colombia	10.8 (21/195)	horse1 (13), D (4), horse2 (4)	[9]
	China	22.5 (75/333)	SC02 (16), horse1 (13), D (1), SCH1(1), SCH3 (1), horse2 (39), YNH1 (1), YNH2 (1), SCH4 (1)	[10]
	China	30.9 (81/262)	EbpC (21), EpbA (20), CS-4 (4), horse1 (4), O (4), G (3), PigEBITS4 (2), CM8 (1), CS-1 (1), CS-4 (1), D (1), ESH-01 (1), Peru8 (1), XJH3 (1), BEB6 (9), CM7 (2), horse2 (2), XJH1 (2), XJH4 (1)	[11]
	Czech R	17.5 (66/377)	D (34), horse1 (7), G (3), EpbA (2), horse11 (2), horse4 (1), horse10(1), WL15 (1), horse2 (8), horse3 (2)	[12]
Donkey	Algeria	1.6 (2/124)	Unknown (2)	[8]
	China	5.3 (16/301)	D (4), NCD-1 (1), NCD-2 (1), J (10)	[13]
Mustang	USA	8.3 (7/84)	horse1 (7)	[12]
Zebra	China	20.0 (1/5)	J (1)	[14]

Previous epidemiological studies have reported the existence of 17 genotypes of *E. bieneusi* in equine animals with only four genotypes (D, NCD-1, NCD-2, and J) in donkeys (Table 3) [8–14]. Among these genotypes, D and J are the most common [8, 13]. Genotype D is a zoonotic genotype and has been reported in more than 20 countries, and isolated from more than 25 animal species, apart from water samples [3, 15, 16]. Genotype J was initially considered to be cattle-based as it was discovered in cattle from China, Argentina, Korea, Germany, the USA, and Portugal [3, 17]. However, recently, this genotype has been discovered in pigs, human, and non-human primates (NHPs) in China [18–20], as well as in other animals, including alpaca, sheep, goat, yak, deer, zebra, bear, and meerkats [3, 21]. The genotype has also been reported in water samples from China [22], in deer from Australia and the USA [23], and in birds from Iran [24]. Until now, genotypes NCD-1 and NCD-2 have only been identified in donkeys [13].

Here, we identified four previously known genotypes (horse1, BEB6, BEB4, and NIAI) and two new genotypes (XJD1 and XJD2), apart from the genotypes D and NCD-2, in donkeys. Genotype horse1 showed good adaptation to equine species as it had only been mentioned in studies performed on horses in China, Colombia, the Czech Republic, Algeria, and the USA [8–12, 14]. Apart from a single case in a NHP [25], we identified this genotype in donkeys in this study. Genotypes BEB4 and BEB6 are mostly found in cattle, humans, and other animals [3, 26]. BEB4 has previously been identified in humans, pigs, and NHPs [18, 19, 27], whilst BEB6 has been found in humans, NHPs, alpacas, deer, goats, sheep, takin, yak, cats, horses, mice, birds, and wastewater [3, 26]. Initially, both BEB4 and BEB6 were discovered in donkeys indicating that their reservoir hosts may be more expansive than predicted. Genotype NIA1 was first identified in an AIDS patient from Nigeria [28], and in an HIV-infected patient from Congo [29]. Until now, none of the studies have reported the presence of genotype NIA1 in any animal species other than humans. This study is the first report on the identification of genotype NIA1 in donkeys confirming potential zoonosis.

It is known that genotypes belonging to groups 1 and 2 represent a large number of hosts, including humans, and may cause most of the zoonotic *E. bieneusi* infections [3]. Here, all genotypes were clustered into either group 1 (horse1, D, NIA1, NCD-2, and XJD2) or group 2 (BEB6, BEB4, and XJD1) (Fig. 1), implying that *E. bieneusi*-infected donkeys could be a potential threat to humans.

## Conclusions

This study first identifies *E. bieneusi* in donkeys in Xinjiang, China. We found a relatively low prevalence of *E. bieneusi* in both farmed and free-ranging donkeys. However, a high level of genetic variation was identified amongst the infected donkeys, with zoonotic genotypes D, BEB6, BEB4, and NIA1. ITS sequencing-based phylogenetic analysis revealed that all the *E. bieneusi* isolates came from donkeys belonging to Groups 1 or 2. These data indicate the possibility of donkey-to-human transmission of this pathogenic parasite.

## Methods

### Sample collection

Six hundred and eighty fecal samples (approximately 50 g each) were gathered from 178 free-ranging donkeys in five countrysides, and 502 farmed donkeys from 18 farms in 13 cities of Xinjiang, China, between May 2016 to December 2018 (Table 1). A sterile disposable latex glove was worn while collecting the fecal samples, immediately post-defecation and placed in labeled sterile bags. Each animal was identified based on neck ropes, ear tags, and body features such as color and size to avoid sample duplication. The collected samples accounted for 10 to 30% of adults or young donkeys on each farm and all free-ranging donkeys in the countryside. The samples were transported under ice-cold conditions and stored at 4 °C until further use. The adult and young donkeys were ≥ 1 year and < 1 year old, respectively. No clinical symptom was observed in any animal at the time of sampling.

### DNA extraction

A suspension was created by mixing the feces sample (approximately 10 g) with 30 mL of distilled water, filtered, and centrifuged for 5 min at 3000 *g*. We used the E.Z.N.A. stool DNA kit to extract the genomic DNA from 200 mg of the fecal samples (pellets), following the manufacturer’s protocol. The isolated DNA (200 μL) from each sample was preserved at − 20 °C before PCR analysis.

### PCR

A 390 bp region of the rRNA gene was amplified using the 2 × EasyTaq PCR SuperMix to examine the prevalence of *E. bieneusi*. Primary and secondary PCR amplifications were performed using two pairs of primers, EBITS1 and EBITS2.4, and EBITS3 and EBITS4, resulting in ~ 390 bp and ~ 435 bp fragments, respectively [30]. Their respective parameters were: 30 cycles of 94 °C for 30 s, 55 °C for 30 s, and 72 °C for 40 s; and 35 cycles of 94 °C for 30 s, 57 °C for 30 s, and 72 °C for 40 s; with a final extension step of 72 °C for 10 min [30]. Distilled water was used as the negative control and DNA from dairy cattle-derived genotype I was used as the positive control.

### Sequencing

GENEWIZ (Suzhou, China) was used to sequence the *E. bieneusi*-positive secondary PCR products. Bi-directional sequencing and the sequencing of PCR products was done to confirm sequence accuracy when required for DNA preparations. The *E. bieneusi* genotypes were detected by sequence alignment using ClustalX v1.83. If the identified genotypes of *E. bieneusi* were similar to the ones in the GenBank database, then they were given the first published name; however, if DNA sequencing confirmed any single nucleotide substitutions/deletions/insertion in the ITS sequences of minimum two PCR products, then they were labeled as new genotypes. All samples were given a genotype identity by adding Arabic numbers after the abbreviation XJD (Xinjiang Donkey) based on the order of appearance. All genotypes were labeled using the established nomenclature system [7].

### Phylogenetic analysis

A neighbor-joining phylogenetic tree was constructed using Mega 7.0 based on the Kimura-2-parameter model to verify the genogroup designation and to evaluate the genetic relationships between the novel identified ITS genotypes of *E. bieneusi* with known sequences. Bootstrap analysis with 1000 replicates was used to assess the reliability of the phylogenetic trees.

## Data Availability

All data generated and/or analyzed during this study are included in this published manuscript. The nucleotide sequences identified in this study were deposited under accession numbers MN174113 to MN174120 in the GenBank database.

## Abbreviations

PCR: Polymerase chain reaction
ITS: Internal transcribed spacer
HIV: Human immunodeficiency virus
NHP: Non-human primates
